# Atherosclerotic Cardiovascular Disease Novel and Traditional Risk Factors in Middle Eastern Young Women. The ANCORS-YW Study

**DOI:** 10.5334/gh.1341

**Published:** 2024-07-15

**Authors:** Ayman J. Hammoudeh, Majeda Jallad, Yousef Khader, Yahya Badaineh, Ramzi A. Tabbalat, Hasan Zammar, Hanna Al-Makhamreh, Asma Basha, Liyan AlAtteili, Raghad Abuhalimeh, Taima Fkheideh, Amr Ababneh, Layan Ababneh, Saad A. Mahmoud, Imad A. Alhaddad

**Affiliations:** 1 Department of Cardiology, 44 Kindi Street, Istishari Hospital, Amman 11184, Jordan; 2 Department of Obstetrics and Gynecology, 44 Kindi Street, Istishari Hospital, Amman 11184, Jordan; 3 Department of Public Health, Jordan University of Science and Technology School of Medicine, 3300 Ramtha Road, Irbid 22110, Jordan; 4 Department of Cardiology, Abdali Hospital, 1 Al-Istethmar Street, Abdali Boulevard, Amman 11191, Jordan; 5 Department of Cardiology, European Gaza Hospital, 12 Khan Yunis Street, Gaza P950, Gaza Strip, Palestine; 6 Department of Internal Medicine, Section of Cardiology, Jordan University Hospital, 100 Queen Rania Street, Amman 11910, Jordan; 7 Department of Obstetrics and Gynecology, Jordan University Hospital, 100 Queen Rania Street, Amman 11910, Jordan; 8 Department of Medical Education, University of Jordan School of Medicine, 100 Queen Rania Street, Amman 11910, Jordan; 9 Department of Medical Education, Jordan University of Science and Technology School of Medicine, Department of Medical Education, 3300 Ramtha Road, Irbid 22110, Jordan; 10 Department of Internal Medicine, King Abdullah University Hospital, 3300 Ramtha Road, Irbid 22110, Jordan; 11 Jordan Cardiovascular Center, Jordan Hospital, 55 Queen Noor Street, Amman 11152, Jordan

**Keywords:** Atherosclerotic cardiovascular disease, Cardiovascular disease in young women, Novel cardiovascular risk factors, Middle Eastern population, Traditional cardiovascular risk factors

## Abstract

**Background:**

There is paucity of data on the prevalence of novel and traditional cardiovascular risk factors in young women with atherosclerotic cardiovascular disease (ASCVD) in the Middle East. We sought to evaluate clinical profiles and prevalence of novel and traditional risk factors in Middle Eastern young women with ASCVD compared with age-matched controls.

**Methods:**

Women 18–50 years of age who have ASCVD were enrolled and each was aged-matched with two women with no ASCVD. Prevalence of novel and traditional risk factors was compared in the two groups. Multivariable analyzes examined the independent association of 16 factors with ASCVD.

**Results:**

Of 627 young women enrolled mean age 44.1 ± 5.2 years; 209 had ASCVD and 418 served as controls. Women with ASCVD had significantly higher prevalence of five of the studied traditional risk factors (hypertension, type 2 diabetes [T2D], smoking, low-density lipoprotein cholesterol serum levels, and family history of premature ASCVD [FHx]) than women with no ASCVD. Additionally, of the 11 novel and psychosocial risk factors studied, four showed significantly higher prevalence in young women with ASCVD (preterm delivery, hypertensive disease of pregnancy gestational diabetes, and low level of education). Multivariable analyzes showed hypertension, T2D, smoking, FHx, persistent weight gain after pregnancy and low level of education were independently associated with ASCVD.

**Conclusions:**

In this study of young Middle Eastern women; traditional risk factors as well as persistent weight gain after pregnancy were more prevalent in women with ASCVD compared with controls.

The study is registered with ClinicalTrials.gov, unique identifier number NCT04975503.

## Introduction

Atherosclerotic cardiovascular disease (ASCVD) is the leading cause of death worldwide including the Middle East [1]. It is the most common cause of death in all adult age groups of both sexes, except young, menstruating women who rarely have clinically evident disease and who account for less than 5% of hospitalizations for acute myocardial infarction (AMI) [2]. Young women with acute cardiovascular events represent a unique phenotype associated with a two-fold increase in the risk of in-hospital and one-year mortality compared with men of similar age after adjusting for other clinical variables, especially the traditional risk factors that include hypertension (HTN), type 2 diabetes mellitus (T2D), cigarette smoking, hypercholesterolemia, obesity, physical inactivity, and family history of premature ASCVD (FHx) [2,3]. The recent remarkable decline in the event rates of AMI and its related mortality has not been demonstrated in young women possibly related in part to under-recognition, under-diagnosis, and under-treatment of this group of patients [4].

In addition to the extensively studied role of the traditional risk factors in the development of ASCVD in young women, there is a growing evidence of a strong association of an unfavorable cardiovascular outcome with specific novel risk factors related to events that take place throughout the women’s reproductive age [5]. There is an expanding list of recently-recognized novel risk factors that contribute to premature ASCVD in young women, including distinctive female sex-specific pathological diseases, reproductive age span, menstruation history, pregnancy-related cardiometabolic diseases, malignancy-related complications, autoimmune diseases, and social determinants of health (SDOH) [3,4,5].

Despite these studies, young women, especially in the Middle East, have received little attention related to the prevalence of ASCVD and its novel and traditional risk factors. Studies in this region showed that women, most of whom were postmenopausal, accounted for nearly 20% of patients with ASCVD, and younger women comprising 5% of these patients. The traditional cardiovascular risk factors are highly prevalent in Middle Eastern women with ASCVD, but there are no studies that evaluated age-specific relation of the prevalence and role of novel and traditional risk factors in ASCVD in young women in this region [6]. While investigating traditional risk factors has greatly enhanced the understating of ASCVD and its early diagnosis and management, studying novel risk factors in young women could offer further opportunities for more comprehensive primary and secondary preventive cardiovascular strategies in these women [7]. The Atherosclerotic Cardiovascular Disease Novel and Traditional risk Factors in Middle Eastern Young Women (ANCORS-YW) study is the first to determine the prevalence of novel and traditional cardiovascular risk factors in young women with ASCVD.

## Methods

### Study design

A case-control, multicenter study was conducted in the period between August 2021 and October 2023 in 12 hospitals. Each patient with ASCVD was age-matched (±5 years) with two women controls selected from the same population in each center participating in enrolling case for the study. Specifically, they included health care centers’ workers, visitors and patients’ companions. These women did not have a prior diagnosis of ASCVD, and were free of symptoms suggestive of this disease. The investigators in each center were instructed, once a patient was enrolled, to select two control women fulfilling the above criteria.

Anthropometric and demographic profiles, novel and traditional risk factors, and SDOH (place of residence, level of education, and presence of health insurance) of the patients and controls were documented based on measurements by the interviewing study investigators according to a unified standard protocol.

With a significance level of 0.05, a power of 80%, and assuming a frequency of any one of the studied variables to be 10%, we have calculated the minimum sample size required to detect a clinically significant association (odds ratio = 2) between ASCVD and any of the studied predictors, maintaining a 1:2 case-to-control ratio. The computed sample size was 209 cases and 418 controls.

### Inclusion criteria

Women in the case group were enrolled in the study if they have ASCVD, were aged 18–50 years, were married, had at least one pregnancy, and were willing to sign an informed consent. Women in the control group were enrolled if they fulfilled the same criteria as women in the case group but do not have ASCVD.

### Definition of exposures

ASCVD included acute coronary syndrome (ACS) (ST-segment elevation MI, non-ST-segment elevation MI, and unstable angina), coronary artery disease (CAD) diagnosed by coronary computed tomographic angiography, stroke and transient ischemic attack (diagnosed by a neurologist based on standard clinical and imaging criteria), extracranial carotid artery disease (diagnosed by the presence of atherosclerosis of the common or internal carotid artery evident by arterial Doppler, computed tomographic, or invasive angiography), and peripheral arterial disease (PAD) of the lower extremities (lower extremity ischemic pain and/or atherosclerosis evident by arterial Doppler, computed tomographic, or invasive angiography).

### Definitions of traditional risk factors

Hypertension (HTN) was defined as repeated resting blood pressure (BP) measurements ≥140/90 mm Hg, a prior diagnosis by a treating physician, or use of BP medications. T2D was defined as the presence of classical symptoms of hyperglycemia and casual plasma glucose ≥200 mg/dl, fasting plasma glucose ≥126 mg/dl, serum level of glycated hemoglobin ≥6.5 g/dl, or a prior diagnosis by a treating physician. Dyslipidemia was defined as an elevated serum level of low-density lipoprotein cholesterol (LDL-C) >70 mg/dl in those with ASCVD or T2D, and >116 mg/dl in those with no ASCVD or T2D. Low serum level of high-density lipoprotein cholesterol (HDL-C) was defined as serum levels <50 mg/dl. Body mass index was calculated by the standard formula (weight (kg)/height (m^2^)). Diagnosis of metabolic syndrome was confirmed by the presence of at least three of the following criteria: HTN, obesity (BMI ≥ 30 kg/m^2^), serum level of HDL-C <50 md/dl, and serum level of triglycerides >150 mg/dl.

### Definitions of novel risk factors

Preterm delivery was defined as a live delivery before 37 weeks and after 20 weeks of gestation. Hypertensive disorders of pregnancy (HDP) was defined as any of gestational HTN (occurring for the first time after 20 weeks of gestation with no significant proteinuria or other biochemical or hematological abnormalities), chronic HTN (diagnosed before the 20th week of gestation), pre-eclampsia defined as HTN after the 20th week of gestation associated with significant proteinuria or evidence of other biochemical or hematological abnormalities) and eclampsia (defined as seizures not attributable to other causes in the presence of preeclampsia).

Gestational diabetes mellitus (GDM) was diagnosed if one or more of the following criteria are met: fasting plasma glucose ≥126 mg/dl, 2-h plasma glucose ≥200 mg/dl following a 75 g oral glucose load, and random plasma glucose ≥200 mg/dl in the presence of diabetes symptoms.

Weight gain after pregnancy was defined according to the Institute of Medicine criteria [8] and refers to weight retention 12 months after delivery as gaining more than 16 kg in women with a normal pre-pregnancy BMI, gaining more than 11 kg in those who were overweight prior to pregnancy, or gaining more than 9 kg in those who were obese prior to pregnancy.

Polycystic ovary syndrome (PCOS) was defined by the presence of two clinical or biochemical hyperandrogenism features, ovulatory dysfunction, or polycystic ovaries. Premature menopause was defined as oligo-amenorrhea of more than 12 months associated with serial elevated gonadotropins on three occasions measured 4–6 weeks apart in women under the age of 40 years. Depression was defined as prior diagnosis by a psychiatrist, or prescription of antidepressant medication.

The centers that participated in the study enrolled equal proportion (8–11%) of the whole cohort. The definition of ASCVD was uniform and was used by all centers. Although treatment of ASCVD was according to discretion of the treating cardiologist, such therapeutic strategies followed the current practice guidelines. All of the participating hospitals were tertiary care centers with 24/7 cardiac catheterizations services.

This non-interventional study has been performed in accordance with the Declaration of Helsinki. The study received proper ethical oversight and Institutional Review Board approval from the participating institutions (Institutional Review Board/Independent Ethics Committee Istishari hospital, Amman, Jordan, Approval number IH-IRB-IRC-7-29-2021). Each patient signed a written informed consent. The study is registered with ClinicalTrials.gov (NCT04975503).

### Statistical analysis

Data were analyzed using IBM SPSS Statistics version 24. Descriptive statistics were performed using means and standard deviation (SD) to describe the continuous variables and proportions to describe the categorical variables. Independent t-test was used to compare means and chi-square test was used to compare percentages of the variables in women with ASCVD and those who have no ASCVD. Binary logistic regression analysis was conducted to determine factors associated with ASCVD. A p-value of less than 0.05 was considered statistically significant.

### Results

Of the 627 women enrolled, 209 had ASCVD, and 418 served as controls. Comparison of the anthropometric, demographic, socioeconomic and clinical features of the ASCVD and control groups are shown in Table 1. The majority (eight in ten) of women in both groups were 40–50 years of age. Women in the ASCVD group were less likely to have received graduate or post graduate education. More women with ASCVD had health insurance compared with the control group. Urban or rural residence did not significantly differ in both groups.

**Table 1:** Baseline demographic, socioeconomic, and clinical characteristics of 624 young women with or without ASCVD.


VARIABLE	ASCVD GROUP N = 209 (n,%)	CONTROL GROUP N = 418 (n,%)	p-VALUE

Demographic and socioeconomic status, N (%)			

Mean age ± SD (years)	44.8 ± 5.2	43.8 ± 5.2	0.024

Age strata			0.312

18–40 years	37 (17.7)	90 (21.5)	

41–50 years	172 (82.3)	328 (78.5)	

Race			

Caucasian Arab	209 (100)	418 (100)	–

Residence			

Capital city	78 (37.3)	236 (56.4)	<0.001

Urban	315 (75.4)	148 (70.8)	0.263

Rural	103 (24.6)	61 (29.2)	0.263

Education			<0.001

None, school, and diploma	156 (74.6)	231 (55.3)	

Graduate and postgraduate education	53 (25.4)	187 (44.7)	

Health insurance	149 (71.3)	238 (56.9)	<0.001

Traditional risk factors, N (%)			

Hypertension	118 (56.5)	114 (27.3)	<0.001

Type 2 diabetes mellitus	83 (39.7)	49 (11.7)	<0.001

Elevated serum level of LDL-C	114/129 (88.4)	86/122 (70.5)	<0.001

Low serum level of HDL-C	96/122 (78.7)	63/114 (55.3)	<0.001

Family history of premature ASCVD	101 (48.3)	98 (23.4)	<0.001

Smoking			

Current cigarette smoking	69 (33.0)	89 (21.3)	0.002

Past cigarette smoking	13 (6.2)	12 (2.9)	0.076

Current water pipe smoking	9 (4.3)	41 (9.8)	0.025

Current electronic cigarette smoking	4 (1.9)	12 (2.9)	0.632

All current smokers	82 (39.2)	142 (34.0)	0.233

Second-hand smoking	38 (18.2)	86 (20.6)	0.545

Current cigarette smoking			0.002

≤10 pack.years	36/69 (52.2)	69/89 (77.5)	

>10 pack.years	33/69 (47.8)	20/89 (22.5)	

Body mass index (kg/m^2^)			0.361

<24.9	46 (22.0)	97 (23.2)	

25–29.9	68 (32.5)	147 (35.2)	

≥30	95 (45.5)	174 (41.6)	

Metabolic syndrome	83/160 (51.9)	57/178 (32.0)	<0.001

Regular physical activity			0.279

Yes	47 (22.5)	110 (26.3)	

No	162 (77.5)	308 (73.7)	

Exercise duration per week			0.077

<3 hours	6/47 (12.8)	30/110 (27.3)	

3–7 hours	30/47 (63.8)	69/110 (62.7)	

>7 hours	11/47 (23.4)	11/110 (10.0)	

Novel risk factors, N (%)			

Preterm delivery	55 (26.3)	70 (16.7)	0.006

Hypertensive disease of pregnancy			

All	64 (30.6)	95 (22.7)	0.041

Gestational HTN	25 (12.0)	53 (12.7)	0.903

Chronic HTN	20 (9.6)	9 (2.2)	<0.001

Preecalmpsia	19 (9.1)	33 (7.9)	0.719

Gestational DM	35 (16.7)	43 (10.3)	0.031

Persistent weight gain after pregnancy	31 (14.8)	89 (21.3)	0.053

Polycystic ovary syndrome	14 (6.7)	28 (6.7)	0.865

Premature menopause	20 (9.6)	42 (10.0)	0.987

Breast cancer	1 (0.5)	4 (0.1)	0.920

Depression	18 (8.6)	36 (8.6)	0.880

Autoimmune/collagen vascular disease	14 (6.7)	17 (4.1)	0.223

Other comorbid diseases and procedures, N (%)			

Metabolic syndrome	63/160 (51.9)	57/178 (32.0)	<0.001

Heart failure	26 (12.4)	2 (0.5)	<0.001

Chronic kidney disease	6 (2.9)	2 (0.5)	0.033

Sleep apnea	6 (2.9)	2 (0.5)	0.033

Hypercoagulable state and VTE	6 (2.9)	4 (1.0)	0.151

Thyroid disease	21 (10.0)	56 (13.4)	0.274

Malignancy	3 (1.4)	7 (1.7)	0.956

Bilateral salpingo-oopherectomy	3 (1.4)	10 (2.4)	0.595

Caesarian section	21 (10.0)	88 (21.1)	<0.001


ASCVD: atherosclerotic cardiovascular disease; DM: diabetes mellitus; HTN: hypertension; HDL-C: high-density lipoprotein cholesterol; LDL-C: low-density lipoprotein cholesterol; SD: Standard deviation; VTE: Venous thromboembolism.

**Figure 1:**
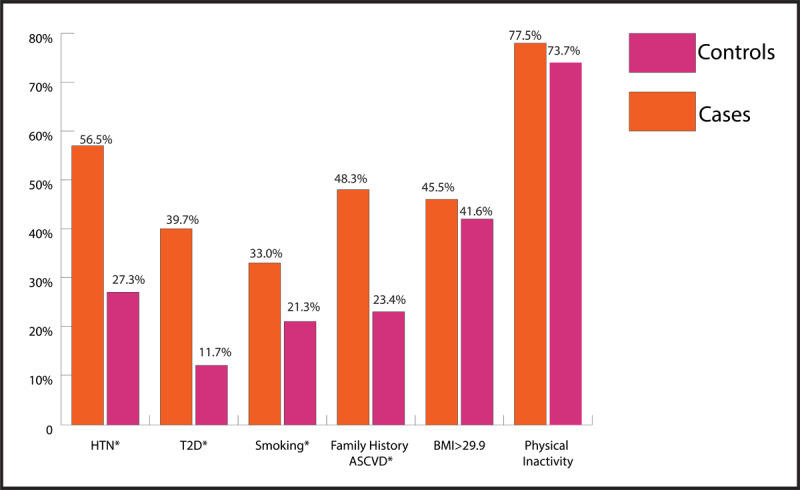
ASCVD Risk Factors in Middle Eastern Young Women. *p < 0.05. HTN: hypertension, T2D: Type 2 diabetes, BMI: Body Mass Index, ASCVD: Atherosclerotic cardiovascular disease.

Five traditional risk factors (HTN, T2D, cigarette smoking, FHx, and elevated LDL-C) were more prevalent in young women with ASCVD than the control group (Table 1 and Figure 1). Serum levels of LDL-C and HDL-C were available for 62% and 29% of the ASCVD and control groups, respectively. Mean LDL-C in the ASCVD group was 112 ± 34.7 mg/dl, and 10.0% of those had an LDL-C of >190 mg/dl. High levels of LDL-C and low levels of HDL-C were more prevalent in the ASCVD group. Prevalence rates of obesity and physical inactivity were not different between the two groups.

**Figure 2:**
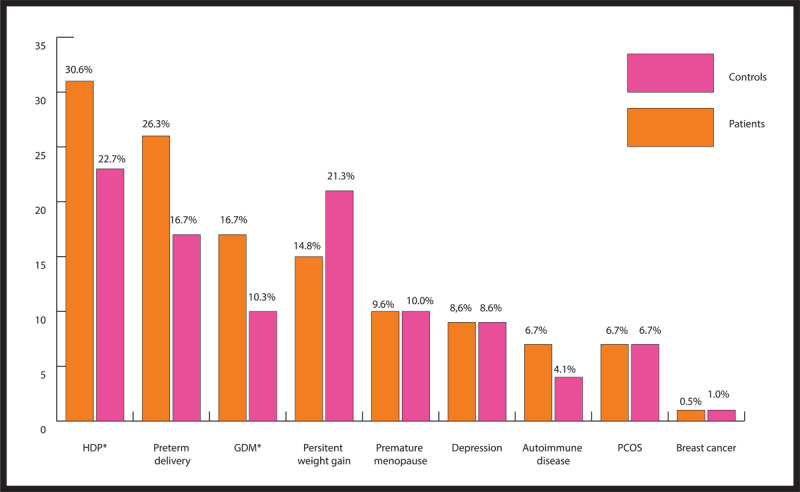
Novel risk factors for atherosclerotic cardiovascular disease in young women with or without disease. *p < 0.05. HDP: Hypertension of pregnancy, GDM: Gestational diabetes, PCOS: Polycystic ovary syndrome.

Of all novel risk factors studied (Table 1 and Figure 2), preterm delivery, HDP, and GDM were more prevalent in the ASCVD group than the control group. The higher prevalence of HDP in women with ASCVD was mainly driven by chronic HTN, rather than preeclampsia and gestational HTN. No significant differences were demonstrated between the two groups in the prevalence rates of premature menopause, PCOS, breast cancer, depression, or autoimmune disease.

Details of the diagnosed ASCVD are shown in Table 2. The majority (86%) of patients had CAD, and 95% of those had ACS too. Percutaneous or surgical coronary revascularization was undertaken in around 70% of those who had ACS. Around one in five women had a stroke, and only 8% had multiarterial bed involvement (≥2 of the coronary, carotid, and peripheral arterial disease).

**Table 2:** Classification and details of atherosclerotic cardiovascular disease in 209 young women.


DISEASE	N	%

Coronary artery disease	180	86.1

Acute coronary syndrome:		

All	171	81.8

ST-segment elevation myocardial infarction	56/171	33.3

Non-ST-segment elevation acute coronary syndrome	115/171	67.3

Obstructive coronary artery disease	118/180	65.6

Non-obstructive coronary artery disease	62/180	34.4

Coronary revascularization in acute coronary syndrome:		

All	118/171	69.0

Percutaneous coronary angioplasty	113/171	66.1

Coronary artery bypass graft surgery	5/171	2.9

Spontaneous coronary artery dissection	6/180	3.3

Coronary artery disease by computed tomography	9/180	5.0

Transient ischemic attack/Stroke	40	19.1

Peripheral artery disease	6	2.9

Multiarterial beds involvement	17	8.1


ACS: acute coronary syndrome; CABG: coronary artery bypass graft; CAD: coronary artery disease; CCTA: coronary computed tomography angiography; NSTEMI: non-ST-segment elevation myocardial infarction; SCAD: spontaneous coronary dissection; STEMI: ST-segment elevation myocardial infarction.

Prescribed medications for both groups are shown in Table 3. All secondary preventive medications, including oral antiplatelet agents, statins, and beta blocker were prescribed for the majority, and renin-angiotensin system inhibitors for nearly half of the patients with ASCVD. The mean blood pressure (mmHg) readings in the patient and control groups at baseline were 133/81 vs. 131/79, respectively, p = 0.15. Compared to women in the control group, more women with ASCVD were prescribed one antihypertension medication (37.8% vs. 23.7%, p < 0.001), or two medications (38.3% vs. 5.5%, p < 0.002), respectively. Three medications were prescribed in similar proportions for both groups (6.2% vs. 3.3%, p = 14).

**Table 3:** Use of medications in young women with atherosclerotic cardiovascular and the control group.


MEDICATION	ASCVD GROUP N = 209	CONTROL GROUP N = 418	p-VALUE

Oral antiplatelet agents:			

One agent	76 (36.4)	36 (8.6)	<0.001

Aspirin and a second agent	112 (53.6)	2 (0.5)	<0.001

Oral anticoagulant agents	9 (4.3)	6 (1.4)	0.048

Lipid lowering agents:			

All statins	187 (89.5)	65 (15.6)	<0.001

High intensity statin	116/187 (62.0)	28/65 (43.1)	0.012

Statin/ezetimibe combination	8/187 (4.3)	5/65 (7.7)	0.459

Beta blockers	152 (72.7)	89 (21.3)	<0.001

Renin angiotensin system inhibitors	101 (48.3)	68 (16.3)	<0.001

Diuretics	31 (14.8)	32 (7.7)	0.008

Antidiabetic agents	65 (31.1)	38 (9.1)	<0.001


Multivariable analyzes utilizing 17 variables (6 traditional risk factors, and 11 novel and psychosocial risk factors), demonstrated that four traditional (HTN, T2D, FHx, and cigarette smoking), and one novel risk factor (persistent weight gain after pregnancy) were independently associated with ASCVD (Supplement Table and Table 4). The Multivariable analyzes did not include LDL-C or HDL-C (and subsequently, metabolic syndrome) because lipoprotein cholesterol serum levels were not available for all participating women.

**Table 4:** Multivariate analysis of factors associated with ASCVD in young women.


	ODDS RATIO	95% CONFIDENCE INTERVAL		p-VALUE

Low level of education	1.81	1.18	2.76	0.006

Urban residency	0.90	0.57	1.40	0.629

No health insurance	0.50	0.33	0.77	0.001

Hypertension	2.15	1.41	3.30	<0.000

Type 2 diabetes	2.97	1.83	4.82	<0.000

Family history of premature atherosclerotic cardiovascular disease	2.68	1.80	4.00	<0.000

Body mass index ≥ 30.0 kg/m^2^	0.88	0.59	1.33	0.554

Current cigarette smoking	2.02	1.30	3.15	0.002

Physical inactivity	0.96	0.61	1.52	0.868

Preterm delivery	1.59	0.98	2.56	0.060

Hypertension disease of pregnancy	1.19	0.75	1.87	0.464

Gestational diabetes mellitus	1.19	0.66	2.14	0.567

Persistent weight gain after pregnancy	0.57	0.34	0.97	0.040

Autoimmune disease	1.73	0.73	4.12	0.216

Breast cancer	0.40	0.04	4.24	0.445

Depression	0.95	0.46	1.94	0.884

Premature menopause	1.00	0.53	1.92	0.989

Polycystic ovary syndrome	0.78	0.34	1.77	0.547


## Discussion

To our knowledge, this is the most comprehensive and contemporary assessment of the prevalence of traditional and novel cardiovascular risk factors in Middle Eastern young women with ASCVD compared with age-matched controls. The major finding from this study is that most of the traditional risk factors had significantly higher prevalence rates among women with ASCVD compared with the controls, and these risk factors were independently associated with ASCVD by multivariate analysis. Prevalence of novel risk factors, on the other hand, was inconsistent, and despite higher prevalence of several of these factors in women with ASCVD, only one of them was independently associated with ASCVD.

The assumption that young women are immune to ASCVD, and that it is difficult to control the underlying risk factors of this disease has been challenged by recent clinical and epidemiological studies [2,3,4,9]. Studies of risk profiles and pathophysiology of ASCVD in young women demonstrated a long list of risk factors that can be diagnosed and treated early in order to curtail the observed unfavorable outcome in these women compared with men in the same age [10]. Women comprise about half of the 11.3 million population in Jordan, and young women (18–50 years of age) account for 20.8% of these, a proportion larger than that seen in western countries. Most of the young women with ASCVD in this study were in their forties of age, and the majority had CAD and presented as ACS. Additionally, most ACS patients were treated with contemporary management, including coronary revascularization, and proper secondary cardiovascular prevention pharmacotherapy, including dual oral antiplatelet agents, statins, and beat blockers.

### Traditional risk factors

Not unexpectedly, the study demonstrated a robust association between most of traditional risk factors and ASCVD in the population under consideration. Global cohorts of women and men showed that significant portion of incident ASCVD and all-cause deaths may be attributable to traditional risk factors [1,2,3]. In the Middle East, traditional risk factors, including HTN, T2D, cigarette smoking, obesity, dyslipidemia, and FHx have stronger association with ASCVD in both sexes of all age groups, and our study confirmed this association in young women as well [7,11,12].

In addition to the fact that traditional risk factors were more prevalent in young women with ASCVD, the study demonstrated an alarmingly high prevalence rates of these risk factors in young women in the control group as well. These rates are higher than those reported from western and South East Asian populations, particularly HTN, T2D, LDL-C levels, cigarette smoking, obesity, and physical inactivity [12,13]. Physical inactivity and sedentary behaviors remain a worldwide concern despite the proven cardioprotective role of regular exercise in the primary and secondary prevention of ASCVD [14]. The majority of women in the current study in both the ASCVD and control groups did not exercise on regular basis, probably a reflection of social factors that might limit widespread participation of women in outdoors physical activities in some local communities.

### Novel risk factors

The list of sex-specific risk factors shown to have strong association with ASCVD in young women is large and expanding. It includes, in addition to the risk factors examined in the current study, early menarche, intrauterine growth retardation, abortion, still birth, earlier age at first birth, low- or high-birth weight fetus, short reproductive life span, hypothalamic amenorrhea, oral contraceptives, and hormone replacement [2,4,5,15]. The current study demonstrated a significantly higher prevalence of three novel risk factors in women with ASCVD (HDP, GDM, and preterm delivery), and only one other risk factor (persistent weight gain after pregnancy) being independently associated with ACSV on multivariate analysis.

Approximately 30% of women experience an adverse pregnancy outcome (APO), including HDP, preterm delivery, and GDM [16]. The long term (7–10 years) association of multiple novel risk factors with ASCVD was ≥2-fold or greater for pre-eclampsia, GDM, and preterm birth; 1.5–1.9-fold for premature ovarian insufficiency, and early menopause; and <1.5-fold for PCOS [9,15,16]. Despite the potential influence by residual confounding on the association of APO with ASCVD, recent evidence has demonstrated a causal genetic relevance of reproductive factors on ASCVD in women [17]. Some authorities advocate considering the sex-specific risk factors to be used for ASCVD risk stratification and prevention programs in women [16].

The current study showed a significantly higher prevalence of HDP in women with ASCVD which was mainly driven by the higher prevalence of chronic HTN, not gestational HTN, or preeclampsia. HDP, and pre-eclampsia in particular, is a major cause of maternal and perinatal morbidity and mortality, and has a fourfold and threefold increased risk of AMI and stroke, respectively, than women without pre-eclampsia ten years after delivery [18]. Multiple pathophysiological mechanisms might explain this association, including a high inflammatory milieu, anti-angiogenic state, and high blood levels of atherogenic lipids. These changes lead to endothelial dysfunction, lower coronary flow reserve, higher carotid intima-media thickness, subclinical atherosclerosis, arterial stiffness, and accelerated atherosclerosis [19].

The second novel risk factor with significantly higher prevalence in women with ASCVD in this study was GDM. Globally, the prevalence of GDM was estimated at 14.0%, and the prevalence in low-, middle- and high-income countries ranges between 9.2 and 14.2%. There is a two-fold higher risk of subsequent overall cardiovascular diseases in women with a history of GDM than women without GDM. [20]. Pathophysiological plausibility for the relationship between GDM and ASCVD are diverse and includes insulin resistance, elevated levels of LDL-C and triglycerides, low levels of HDL-C, and circulating inflammatory markers which increase the risk of T2D, HTN, and metabolic syndrome [20].

Spontaneous preterm delivery had also significantly higher prevalence in young women with ASCVD than controls. Preterm delivery, which occurs in 5–13% of deliveries globally, is an independent risk factor for long term cardiovascular mortality. It represents a proinflammatory and microvascular disease associated with higher circulating levels of C-reactive protein levels, endothelial dysfunction, genetic mutations of cholesterol metabolism, and subclinical vascular disease. Consequently this leads to 1.4- to 2-fold risk of ASCVD [21].

Persistent weight gain after pregnancy was the only independent novel factor to be associated with ASCVD. Several pregnancy and postpartum weight change trajectories have been identified. After weight gain during pregnancy and immediate weight loss after delivery, some women continue to lose weight, while others regain gestational weight. Several factors lead to failure to restore the pre-pregnancy body weight, such as the maternal age and BMI, parity, dietary changes, lack of time for exercise, decrease in sleep hours, and psychological stress [22].

Premature menopause was reported by ≤10% of the whole cohort in this study, with no significant differences between women with ASCVD and controls. Spontaneous or iatrogenic premature ovarian failure and menopause in young women is associated with a two-fold risk of ASCVD and driven by the diminishing cardioprotective effects of estrogen that include endothelial dysfunction, dyslipidemia, dysglycemia, and metabolic syndrome [23].

This study reported a similar prevalence of PCOS (6.7%) in patients and controls, a rate not different from the global estimated prevalence rate that ranges between 4% and 21% of women before menopause [24]. PCOS is the most common endocrine disorder among women of reproductive age and is associated with several cardiometabolic abnormalities, including obesity, T2D, HTN, and dyslipidemia, and with an increased risk of an increased risk of subclinical atherosclerosis, CAD, and stroke [25]. Recently, Mendelian randomization studies have challenged the causality of PCOS with CAD and stroke [26].

Breast cancer, reported by only 5 (1.2%) women in this study, has been shown by many investigators to increase the risk of ASCVD up to at least two decades especially in women with left breast cancer, those who received high dose of radiation, and those who have classical ASCVD risk factors [27]. The other disease with low incidence in women enrolled in this study was the autoimmune group of disease and reported in 31 (4.9%) women. Systemic inflammatory and autoimmune disorders (including systemic lupus, rheumatoid arthritis, scleroderma, vasculitis, and psoriasis) affect more women than men and are associated with accelerated atherosclerosis likely related to a variety of factors including the coexistence of other risk factors and their duration, lifestyle habits and practices, sex hormone effects, immune dysregulation, and systemic inflammation [28].

### Other risk factors

In addition to the traditional and novel risk factors, certain non-sex-related SDOH and psychological factors have also been linked to ASCVD in young women. SDOH describe the living and working environment of individuals and includes five domains (education, health insurance, neighborhood and environment, economic stability, and social and community context) [29]. The first three of these domains were addressed in the current study. While the great majority of the whole cohort received at least a school education (n = 623, 99.4%), more women in the control group attended college and postgraduate education than women with ASCVD. Studies have demonstrated a negative association between education level and prevalence of ASCVD because higher education level enhances the woman’s ability to make better informed health decisions, seek early and regular health advice, and adhere to prescribed therapies [30]. Concerning the place of residence, we did not find a difference in the place of residence (i.e., urban vs. rural) between the two groups of women. Excess CVD risk burden among urban young women parallels the growing burden of cardiometabolic risk factors such as HTN, T2D, obesity, and physical inactivity [31]. Finally, depression was reported by a similar percentage of patients and controls in this study. Among 35 studies involving about 1 million individuals, depression was associated with increased risk of ASCVD [32].

It is imperative to note that the process of recruiting women in the control group is of paramount importance in this case-control study to ensure reliability of the conclusions reached. The potential biases resulting from the conduction and methodology of the recruitment process of controls may influence the results of the study and their interpretation. Such biases include inaccurate data recall by interviewed women, failure to enroll consecutive patients, and failure to ascertain absence of symptoms that may implicate an undiagnosed ASCVD. Minimizing such biases in the current study relied on the fact that it is a restricted study that involves consecutive women exclusively, and controls were age-matched with patients, and were recruited from the same social environment as that of the patients’. Limiting confounding by utilization of this restriction and selection strategy was enhanced by enrolling more controls (i.e., 2 to 1 matching of controls to patients). Furthermore, the majority of the women in the control group (87.8%) were visitors in the recruiting centers, rather than health care workers (5.3%) or patients’ companions (6.9%). Relying on personal interviews with controls, as opposed to self-reporting questionnaire, further augments the reliability and strengthens the validity of the study conclusions.

## Study limitations

As in all observational studies, unmeasured confounders may still be present despite all strategies discussed above. Information on reproductive life could have recall errors. Possible reverse causality exists where childhood obesity or type 1 DM might lead to reproductive life changes and increased the risk for ASCVD. Data analyzed, irrespective of parity, might have underestimated the prevalence of pregnancy-related events in those with low parity. Finally, the prevalence of the studied traditional and novel risk factors was reported in patients recruited from tertiary care centers and thus may not be fully reflective of all practice settings. Finally, it is worth mentioning that some women in the control group might have had asymptomatic, subclinical ASCVD due to the rather high prevalence rates of certain risk factors, such as metabolic syndrome and HDP among these women. The absence of ASCVD in this study was based on absence of suggestive symptoms rather than performance of cardiovascular imaging modalities to exclude the presence of ASCVD, such as coronary computed tomographic angiography or peripheral arterial Doppler studies.

Despite these limitations, we strongly believe that this study has demonstrated solid findings on the high prevalence of traditional risk factors for ASCVD in young women in this region. The data presented have important implications for cardiovascular health in young women and suggest that a major component of cardiovascular prevention strategies in young women depends heavily on adopting healthy lifestyle and early diagnosis and effective therapy for HTN, T2D, dyslipidemia, LDL-C, obesity, and cigarette smoking. Despite the pleiotropy of associations between novel risk factors and ASCVD in the group studied, comprehensive clinical evaluation of cardiovascular risk in young women should still include detailed reproductive history. Future large-scale studies are needed to focus on assessment of the incremental benefit of adding certain reproductive factors to the commonly utilized cardiovascular risk stratification tools in young women.

## Conclusions

This study explored the prevalence of traditional and novel risk factors in young women with and without ASCVD. Traditional risk factors have a significantly higher prevalence among those with ASCVD than those who do not. These factors remain the major target for curtailing the disease in young women in this region. The emerging role of sex- and pregnancy-specific risk factors, and SDOH should be the next frontier for larger studies.

## Supplement Table

Univariate analysis of ASCVD risk factors

**Table:** 


	WOMEN WITH NO ASCVD N = 418		WOMEN WITH ASCVD N = 209		TOTAL N = 627	p-VALUE

Education						0.000

No school, school, diploma	231	55.3%	156	74.6%	387	

College and post graduate	187	44.7%	53	25.4%	240	

Place of Residence						0.222

Rural	103	24.6%	61	29.2%	164	

Urban	315	75.4%	148	70.8%	463	

Health insurance						0.000

No	180	43.1%	59	28.4%	239	

Yes	238	56.9%	149	71.6%	387	

Hypertension						0.000

No	304	72.7%	91	43.5%	395	

Yes	114	27.3%	118	56.5%	232	

Type 2 diabetes mellitus						0.000

No	369	88.3%	126	60.3%	495	

Yes	49	11.7%	83	39.7%	132	

High serum level of low-density lipoprotein cholesterol						0.000

No	36	29.5%	15	11.6%	51	

Yes	86	70.5%	114	88.4%	200	

Low serum level of high-density lipoprotein cholesterol						0.000

No	50	44.2%	26	21.3%	76	

Yes	63	55.8%	96	78.7%	159	

Family history of premature ASCVD						0.000

No	320	76.6%	108	51.7%	428	

Yes	98	23.4%	101	48.3%	199	

Body mass index (kg/m^2^)						0.361

<30	244	58.4%	114	54.5%	358	

≥30	174	41.6%	95	45.5%	269	

Smoking						0.001

No	329	78.7%	140	67.0%	469	

Yes	89	21.3%	69	33.0%	158	

Regular physical activity						0.297

No	308	73.7%	162	77.5%	470	

Yes	110	26.3%	47	22.5%	157	

Preterm delivery						0.005

No	348	83.3%	154	73.7%	502	

Yes	70	16.7%	55	26.3%	125	

Hypertensive disease of pregnancy						0.032

No	323	77.3%	145	69.4%	468	

Yes	95	22.7%	64	30.6%	159	

Gestational diabetes mellitus						0.021

No	375	89.7%	174	83.3%	549	

Yes	43	10.3%	35	16.7%	78	

Persistent weight after pregnancy						0.053

No	329	78.7%	178	85.2%	507	

Yes	89	21.3%	31	14.8%	120	

Autoimmune disease						0.152

No	401	95.9%	195	93.3%	596	

Yes	17	4.1%	14	6.7%	31	

Breast cancer						0.525

No	414	99.0%	208	99.5%	622	

Yes	4	1.0%	1	0.5%	5	

Depression						1.000

No	382	91.4%	191	91.4%	573	

Yes	36	8.6%	18	8.6%	54	

Premature menopause						0.850

No	376	90.0%	189	90.4%	565	

Yes	42	10.0%	20	9.6%	62	

Polycystic ovary syndrome						1.000

No	390	93.3%	195	93.3%	585	

Yes	28	6.7%	14	6.7%	42	


ASCVD: atherosclerotic cardiovascular disease.

## Data Accessibility Statement

Data, structured methodology, and results are available upon request from the corresponding author (a.hammoudeh@istisharihospital.com).
